# Effect of Pre-fermentative Treatments on Polysaccharide Composition of White and Rosé Musts and Wines

**DOI:** 10.1021/acs.jafc.2c08976

**Published:** 2023-02-25

**Authors:** Leticia Martínez-Lapuente, Zenaida Guadalupe, Manuel Higueras, Belén Ayestarán, Paula Pérez-Porras, Ana Belén Bautista-Ortín, Encarna Gómez-Plaza

**Affiliations:** † Institute of Vine and Wine Sciences, ICVV (University of La Rioja, Government of La Rioja and CSIC), Finca La Grajera, 26007 Logroño, Spain; ‡ Scientific Computation & Technological Innovation Center (SCoTIC), Universidad de La Rioja, 26006 Logroño, Spain; § Department of Food Science and Technology, Faculty of Veterinary Science, University of Murcia, Campus de Espinardo, 30071 Murcia, Spain

**Keywords:** high-power ultrasound, direct pressing, pre-fermentative cold maceration, polysaccharides, white and rosé musts and wines

## Abstract

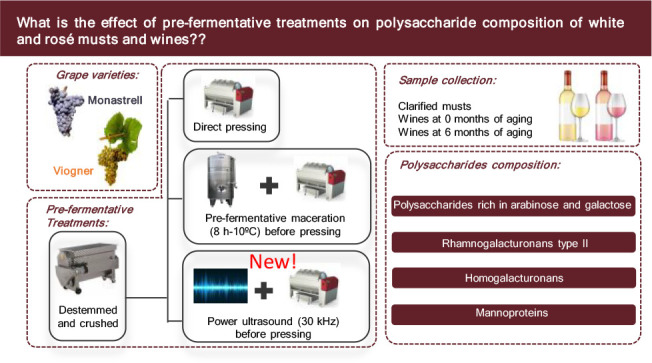

This paper studied the effect of conventional pre-fermentative techniques (direct pressing “CP” and cold maceration “CM”) and an innovate technique (high power ultrasounds “S”), applied to Viogner and Monastrell grapes on the polysaccharide content of the musts, white and rosé wines, and after six months of bottle aging. The results showed that the longer pre-fermentation maceration time applied with the CM technique compared to the short ultrasonic maceration was key in the extraction of polysaccharides from the grape to the must. CP treatment produced wines with the lowest content of total soluble polysaccharide families since it was the least intense pretreatment for the disruption of the grape berry cell wall polysaccharides. Ultrasonic pretreatment could be used as a new tool to increase the solubilization of polysaccharides in wines, positively affecting the wine colloidal properties. During bottle aging, there wasn’t a clear effect of pretreatments on the evolution of polysaccharides.

## 1 Introduction

Polysaccharides are a major group of complex macromolecules present in the wine matrix. Polysaccharides in wine are categorized into two classes; they are either grape or yeast derived. Polysaccharides derived from the pectocellulosic cell walls of grape berries include polysaccharides rich in arabinose and galactose (PRAG), rhamnogalacturonans type II (RG-II), and homogalacturonans (HL).^1−3^ Mannoproteins (MP) are released from yeast cells during fermentation and aging on lees.^1^ In addition, the wine may contain polysaccharides from other sources such as β-glucans from an infection of grapes with *Botrytis cinerea* or exogenous polysaccharides added to wine such as arabic gum or carboxymethylcellulose (CMC). Due to their colloidal nature, polysaccharides interact with other wine components, including aroma compounds, polyphenols, and proteins, and can lead to the modulation of the technological and organoleptic wine quality attributes.^4^ However, not all polysaccharides show the same behavior with respect to wines, and their influence on wine will depend not only on their quantity but also on the type of polysaccharide;^5^ therefore, an understanding of their content and kinetic release is essential.

The transformation of must to wine and later aging in bottle storage produces major changes in the polysaccharide content and composition.^6,7^ In the specific case of white and rosé wines, polysaccharide composition will depend, among other factors, on the pre-fermentative treatment applied to the grapes. The conventional pre-fermentation techniques are the direct pressing applied to avoid the extraction of color, tannin, and herbaceous character in the wine,^8^ and the pre-fermentative maceration of the crushed and destemmed grapes, used to obtain greater color and intensity of the varietal aroma.^9−11^ Several studies have shown that the extraction of polysaccharides into wine can be influenced by the pre-fermentative maceration treatment applied to the grapes.^12−14^ Low temperatures are used during pre-fermentative cold maceration to prevent yeasts from starting alcoholic fermentation; thus, the extraction of grape components can be enhanced in the absence of ethanol.^15^ Therefore, pre-fermentative cold maceration favors the selective diffusion of the water-soluble compounds in aqueous media,^16^ and it allows native endogenous grape pectolytic enzymes to degrade grape cell walls with a prolonged skin contact time prior to alcoholic fermentation, which increases the release and solubilization of grape polysaccharides.^17,18^ However, there are no studies analyzing the effect these treatments on the content of polysaccharides in white and rosé musts or its evolution during wine bottle aging.

Recently, the International Organization of Vine and Wine has officially approved the ultrasound treatment of crushed grapes to promote the extraction of their compounds.^19^ High-power ultrasound (>19 kHz) is a nonthermal technique based on cavitation phenomena, the shock waves created being capable of breaking solid surfaces. For this reason, ultrasounds (US) have been proposed for use in enology to help degrade the cell walls of grape skins, thus facilitating the extraction of the compounds located inside the skin cells.^20^ In fact, it was reported that US facilitate the release of polyphenols and increase the content of some volatile compounds of sensory relevance in red wines, allowing the reduction of maceration time.^21−23^ Since US treatment weakens the cross-linking between pectic and hemicellulosic domains in plant cell walls, an increase of grape polysaccharides in wines from sonicated grapes was also reported.^24,25^ Most studies carried out on US have focused on red wines and have been applied to *Vitis vinifera* L. varieties Monastrell,^20−26^ Cabernet Franc,^27^ Tempranillo,^28^ Tannat,^29^ or Primitivo, Nero di Troia, and Aglianico.^30^ Recently it has been shown that pre-fermentative ultrasound treatment of Viogner grapes increases the aromatic potential in the resulting wines.^31^ However, the contents and extractability of grape components to wines depend on grape characteristics that are influenced by variety; therefore, the US effects may behave differently due to the characteristics of different grape materials.^30,32^

Therefore, the aim of this study was to evaluate, for the first time, the effect of crushed-destemmed grape pressing, cold pre-fermentation maceration, and pre-fermentation sonication of crushed-destemmed grapes on the composition of polysaccharides in *Vitis vinifera* white musts L. cv. Viognier and rosé musts of *Vitis vinifera* L. cv. Monastrell. The evolution of the polysaccharide composition from the must to the wines (at the time of bottling) and after 6 months of bottling was also studied.

## 2 Materials and Methods

### 2.1 Vinification and Sample Collection

White grapes (W) of *var*. Viognier (VIVIC: 13106) and red grapes (R) of *var*. Monastrell (VIVC: 7915) were grown in Jumilla (Murcia, Spain) and were harvested on the vintage 2020 at commercial maturity when they reached 19°Brix and 21°Brix, respectively (hand refractometer ATAGO, Tokyo, Japan).

Grapes (700 kg) were destemmed and crushed (Nouva Zambelli, Saonara Padova, Italy), sulfited (50 mg SO_2_/kg), and divided into three batches. One batch was directly pressed into a pneumatic press (Agritechstore, Trento, Italy) (CP); another was pressed after 8 h at 10 °C of pre-fermentative maceration (CM); and the other was treated with a pilot-scale power ultrasound system (MiniPerseo; Agrovin S.A., Alcazar de San Juan, Spain) using 30 kHz frequency before pressing (S). The US system was applied to the whole batch (300 kg grapes/per hour) and operated at 2500 W with a power density of 8 W/cm^2^.

White musts (WM-CP, WM-CM, WM-S) were then transferred to 50-L stainless-steel tanks by duplication, and a settling step to eliminate small solid parts remaining in suspension was conducted over 24 h at 12 °C, aided with the addition of a pectolytic enzyme preparation (0.4 mL/HL Enozym; Agrovin S.A., Alcazar de San Juan, Spain) to speed up the process by degrading suspended cell wall pectins, therefore reducing must viscosity. After the settling step, musts were racked. Total acidity was corrected to 6 g/L of tartaric acid, and enological nutrients Actimax Natura and Actimax Plus (0.3 g/L; Agrovin, Alcazar de San Juan, España) and commercial *Saccharomyces cerevisiae* yeasts were added to all vinifications (0.20 g/kg, Viniferm BY, Agrovin, Alcazar de San Juan, Spain). When alcoholic fermentation finished (reducing sugars content lower than 2 g/L), total sulfur was corrected to 70 mg/L. Thereafter, the wines were cold-stabilized at 2 °C for one month, racked, and bottled. Samples for analysis were taken of the racked white musts (WM-CP, WM-CM, WM-S), of the young wines when bottling (W0-CP, W0-CM, W0-S), and after 6 months of bottle aging (W6-CP, W6-CM, W6-S). Vinification of red grapes from *Vitis vinifera var*. Monastrell (VIVC: 7915) were made with the same protocol as the white grapes. Samples for analysis were taken of the raked rosé musts (RM-CP, RM-CM, RM-S), of the young wines when bottling (R0-CP, R0-CM, R0-S), and after 6 months of bottle aging (R6-CP, R6-CM, R6-S).

### 2.2 Identification and Quantification of Monosaccharides and Polysaccharides Families by GC–MS

Must and wine polysaccharides were recovered by precipitation after ethanolic dehydration as previously described.^3,5^ The monosaccharide composition was determined by GC–MS of their trimethylsilyl-ester *O*-methyl glycosyl residues obtained after acidic methanolysis and derivatization as previously described.^5^ Total monosaccharides of the precipitated polysaccharides were called TMS. The content of each polysaccharide family was estimated from the concentration of individual glycosyl residues that are characteristic of structurally identified must and wine polysaccharides.^1,5,33,34^ PRAG were estimated from the sum of galactosyl, arabinosyl, rhamnosyl, and glucuronosyl residues; all the mannose content in wines was attributed to yeast mannoproteins; the RG-II content was calculated from the sum of its diagnostic monosaccharides, which represent approximately 25% of the RG-II molecule. Considering the molar ratios of the RG-II (1 residue of 2-O-methyl fucose, 3.5 rhamnose, 2 arabinose, 2 galactose, 1 glucuronic acid, and 9 galacturonic acid), the remaining part was attributed to the presence of PRAG in the case of rhamnose, arabinose, galactose, and glucuronic acid. The remaining galacturonosyl residues were used to estimate the content of homogalacturonans (HL).^5,35^ The content of total soluble polysaccharide families (TSP) was estimated from the sum of MP, RG-II, HL, and PRAG.^3,33^ Four replicates of analysis were performed for each wine sample.

### 2.3 Statistical Analyses

Statistical analyses were performed using SPSS data analysis statistics software system version 15.0 (SPSS Inc., Chicago, IL, USA) and the XLstat-Pro (Addinsoft, Paris, France) program. Both Kolgomorov-Smirnov and Levene’s tests respectively rejected the normality and the homoscedasticity assumptions for most of the monosaccharides and polysaccharide’s levels. Consequently, a nonparametric analog to analysis of variance (ANOVA), the Kruskal–Wallis test, and a nonparametric equivalent to the independent samples *t* test, the Mann–Whitney test, were conducted on raw data. A significance level of 0.05 was considered; thus, the results of the tests were determined statistically significant for *p*-values lower than 0.05.

## 3 Results and Discussion

### 3.1 Effect of Pre-fermentative Treatment on Monosaccharide Composition and Polysaccharide Families of White and Rosé Musts and Wines

Tables 1 and 2 show the monosaccharide composition of polysaccharides and polysaccharide families in white and rosé musts and wines during the bottle aging.

**Table 1:** Monosaccharide Composition (mg/L) of Polysaccharides and Polysaccharides Families in White Musts and Wines

	must^c^			0 months of aging^c^			6 months of aging^c^		
parameter^a^	WM-CP	WM-CM	WM-S	W0-CP	W0-CM	W0-S	W6-CP	W6-CM	W6-S
2-*O*MeFuc^b^	0.16 ± 0.03 a,A	0.33 ± 0.00 b,B	0.43 ± 0.02 c,A	0.60 ± 0.07 a,B	0.54 ± 0.02 a,C	1.08 ± 0.10 a,C	0.57 ± 0.01 b,B	0.20 ± 0.02 a,A	0.54 ± 0.04 b,B
2-*O*MeXyl^b^	0.04 ± 0.01 a,A	0.12 ± 0.01 b,A	0.17 ± 0.04 b,A	0.33 ± 0.00 a,C	0.34 ± 0.00 b,B	0.32 ± 0.04 ab,B	0.14 ± 0.02 a,B	0.15 ± 0.02 a,A	0.32 ± 0.03 b,B
Api^b^	0.20 ± 0.01 b,B	0.13 ± 0.01 a,A	0.16 ± 0.05 b,A	0.16 ± 0.05 a,AB	0.23 ± 0.02 a,B	0.26 ± 0.03 a,B	0.15 ± 0.02 a,A	0.16 ± 0.04 a,A	0.12 ± 0.03 a,A
Ara^b^	18.98 ± 1.02 a,C	23.08 ± 0.77 b,C	25.35 ± 0.20 c,C	8.45 ± 0.76 a,B	11.01 ± 1.91 a,B	12.13 ± 0.43 a,B	2.33 ± 0.36 a,A	2.19 ± 0.09 a,A	3.33 ± 1.01 a,A
Rha^b^	6.28 ± 0.31 a,B	6.35 ± 0.38 a,B	7.43 ± 1.90 a,B	2.39 ± 0.48 a,A	3.33 ± 0.52 a,A	6.00 ± 0.49 b,B	2.91 ± 0.50 a,A	2.98 ± 1.27 ab,A	3.57 ± 0.04 b,A
Fuc^b^	1.28 ± 0.04 b,B	0.65 ± 0.21 a,B	1.52 ± 0.21 b,B	0.35 ± 0.02 a,A	0.37 ± 0.08 a,A	0.57 ± 0.08 b,A	0.35 ± 0.00 a,A	0.37 ± 0.13 a,AB	0.40 ± 0.09 a,A
Xyl^b^	5.49 ± 0.29 a,C	7.50 ± 1.23 b,B	8.69 ± 0.47 b,C	2.32 ± 0.42 a,B	2.03 ± 0.20 a,A	2.68 ± 0.82 a,B	1.29 ± 0.11 a,A	1.60 ± 1.39 a,A	1.27 ± 0.32 a,A
Man^b^	9.21 ± 0.80 a,A	9.15 ± 0.26 a,A	13.39 ± 1.73 b,A	232.42 ± 26.69 a,C	269.72 ± 7.87 b,C	268.76 ± 32.40 ab,C	120.68 ± 24.26 a,B	220.95 ± 1.90 c,B	173.72 ± 4.58 b,B
Gal^b^	178.60 ± 6.29 a,C	253.71 ± 7.93 c,C	205.35 ± 16.69 b,C	59.73 ± 4.12 a,B	65.17 ± 1.40 a,B	74.48 ± 2.19 b,B	34.54 ± 4.56 a,A	55.40 ± 3.84 b,A	35.96 ± 5.97 a,A
GalA^b^	17.43 ± 0.31 a,C	24.93 ± 7.31 a,C	27.66 ± 15.86 a,AB	8.23 ± 1.49 a,A	8.80 ± 0.17 a,B	25.20 ± 2.28 b,B	11.06 ± 0.58 b,B	2.94 ± 0.22 a,A	12.31 ± 2.27 b,A
GluA^b^	7.22 ± 0.22 a,B	9.50 ± 2.28 a,B	9.95 ± 2.60 a,B	1.37 ± 0.13 a,A	1.38 ± 0.12 a,A	1.56 ± 0.30 a,A	0.98 ± 0.26 a,A	1.55 ± 0.21 b,A	1.51 ± 0.35 ab,A
Kdo^b^	0.57 ± 0.26 a,B	0.38 ± 0.03 a,B	0.80 ± 0.59 a,B	0.17 ± 0.04 a,A	0.44 ± 0.06 b,B	0.56 ± 0.21 b,B	0.18 ± 0.07 ab,A	0.25 ± 0.01 b,A	0.12 ± 0.01 a,A
Glc^b^	2603.81 ± 253.69 a,C	5327.28 ± 36.15 c,C	4630.27 ± 470.45 b,C	28.02 ± 2.04 a,B	35.31 ± 2.11 b,B	37.00 ± 1.11 b,B	16.56 ± 4.71 ab,A	22.02 ± 1.84 b,A	12.28 ± 1.01 a,A
TMS – (Glc)	245.45 ± 6.23 a,B	335.85 ± 3.27 b,B	300.91 ± 39.94 b,A	316.52 ± 19.24 a,C	363.36 ± 9.21 b,C	393.60 ± 36.93 b,C	175.18 ± 18.18 a,A	288.74 ± 8.57 c,A	233.17 ± 1.82 b,B
TMS – (Glc + Man + Xyl)	230.76 ± 5.96 a,C	319.19 ± 4.02 b,C	278.82 ± 37.73 b,C	81.78 ± 10.56 a,B	91.61 ± 2.59 a,B	122.16 ± 4.32 b,B	53.22 ± 6.19 a,A	66.18 ± 5.27 b,A	58.17 ± 5.69 ab,A
TMS^b^	2849.27 ± 259.93 a,C	5663.13 ± 32.88 c,C	4931.17 ± 510.38 b,C	344.54 ± 17.20 a,B	398.68 ± 7.11 b,B	430.60 ± 38.04 b,B	191.75 ± 22.89 a,A	310.76 ± 10.40 c,A	245.45 ± 0.81 b,A
Ara/Gal	0.13 ± 0.00 b,B	0.11 ± 0.01 a,B	0.15 ± 0.01 c,A	0.17 ± 0.00 a,C	0.20 ± 0.04 ab,C	0.20 ± 0.00 b,B	0.08 ± 0.00 b,A	0.05 ± 0.00 a,A	0.12 ± 0.05 b,A
Rha/GalA	0.43 ± 0.03 a,B	0.32 ± 0.11 a,A	0.35 ± 0.12 a,A	0.34 ± 0.01 b,A	0.45 ± 0.06 c,A	0.28 ± 0.00 a,A	0.31 ± 0.04 a,A	1.22 ± 0.60 b,B	0.35 ± 0.07 a,A
(Ara + Gal)/Rha	29.21 ± 0.35 a,B	40.41 ± 1.40 b,B	29.61 ± 5.48 a,C	27.03 ± 3.49 b,B	21.70 ± 316 b,A	13.55 ± 0.69 a,B	11.75 ± 0.47 a,A	19.19 ± 6.93 b,A	10.22 ± 1.33 a,A
RG-II^b^	5.63 ± 0.72 a,A	12.71 ± 0.14 b,B	16.53 ± 1.36 c,A	24.56 ± 2.26 a,C	22.79 ± 0.55 a,C	39.81 ± 2.43 b,C	20.44 ± 0.53 b,B	8.75 ± 0.42 a,A	22.40 ± 1.71 b,B
HL^b^	16.01 ± 0.57 a,C	21.92 ± 7.28 a,C	23.76 ± 15.64 a,AB	2.81 ± 0.87 a,A	3.94 ± 0.01 b,B	15.46 ± 3.14 c,B	5.96 ± 0.52 ab,B	1.15 ± 0.41 a,A	7.44 ± 2.62 b,A
PRAG^b^	255.87 ± 9.33 a,C	358.99 ± 9.75 c,C	296.47 ± 22.19 b,C	84.79 ± 5.99 a,A	94.75 ± 0.00 b,B	106.51 ± 3.53 c,B	45.91 ± 6.34 a,B	74.71 ± 5.19 b,A	48.29 ± 7.15 a,A
MP^b^	11.51 ± 1.00 a,A	11.44 ± 0.33 a,A	16.74 ± 2.17 b,A	290.53 ± 33.36 a,C	337.15 ± 9.84 b,C	335.96 ± 40.50 ab,C	150.85 ± 30.32 a,B	276.19 ± 2.38 c,B	217.15 ± 5.73 b,B
TSP^b^	289.02 ± 8.48 a,B	405.05 ± 2.27 c,B	353.50 ± 41.36 b,B	402.69 ± 24.24 a,C	458.63 ± 10.39 b,C	497.74 ± 44.74 b,C	223.15 ± 22.93 a,A	360.80 ± 7.57 c,A	295.28 ± 2.34 b,A

a Average of the two measurements. Different letters show statistically significant differences as obtained by Kruskal–Wallis (α = 0.05) with Mann–Whitney pairwise comparison. Lowercase letters compare treatments in the same stage of winemaking. Uppercase letters compare samples of the same treatment in different stages of winemaking.

b 2-*O*MeFuc, 2-*O*-CH_3_-fucose; 2-*O*MeXyl, 2-*O*-CH_3_-xylose; Api, apiose; Ara, arabinose; Rha, rhamnose; Fuc, fucose; Xyl, xylose; Man, mannose; Gal, galactose; GalA, galacturonic acid; GluA, glucuronic acid, Kdo, 2-keto-3-deoxyoctonate ammonium salt, Glc, glucose; TMS, total monosaccharides; RG-II, rhamnogalacturonans type II; HL, homogalacturonans; PRAG, polysaccharides rich in arabinose and galactose; MP, mannoproteins; TSP, total soluble polysaccharide families.

c WM, white must; W0, white wine at 0 months of aging; W6, white wine at 6 months of aging; CP: direct pressing; CM: pre-fermentation maceration; S: pre-fermentative sonication.

**Table 2:** Monosaccharide Composition (mg/L) of Polysaccharides and Polysaccharide Families in Rosé Musts and Wines^c^

	must^c^			0 months of aging^c^			6 months of aging^c^		
parameter^a^	RM-CP	RM-CM	RM-S	R0-CP	R0-CM	R0-S	R6-CP	R6-CM	R6-S
2-*O*MeFuc^b^	0.09 ± 0.01 a,A	0.23 ± 0.00 c,A	0.12 ± 0.00 b,A	0.48 ± 0.07 a,B	1.53 ± 0.30 b,B	1.39 ± 0.34 b,C	0.48 ± 0.10 a,B	1.20 ± 0.31 b,B	0.50 ± 0.03 a,B
2-*O*MeXyl^b^	0.10 ± 0.02 a,A	0.18 ± 0.02 b,A	0.12 ± 0.02 a,A	0.26 ± 0.06 a,B	0.84 ± 0.23 b,C	0.94 ± 0.04 b,C	0.21 ± 0.12 a,AB	0.51 ± 0.01 c,B	0.40 ± 0.01 b,B
Api^b^	0.03 ± 0.01 a,A	0.04 ± 0.00 a,A	1.09 ± 0.20 b,B	0.12 ± 0.05 a,B	0.49 ± 0.19 b,C	0.31 ± 0.06 b,A	0.14 ± 0.07 ab.B	0.12 ± 0.02 a,B	0.22 ± 0.04 b,A
Ara^b^	15.00 ± 1.89 a,C	46.37 ± 7.70 b,C	58.50 ± 7.42 b,C	6.28 ± 0.13 a,B	6.80 ± 0.87 a,B	6.46 ± 1.92 a,B	1.71 ± 0.48 a,A	2.48 ± 0.43 a,A	2.16 ± 0.52 a,A
Rha^b^	3.34 ± 1.41 a,A	7.61 ± 1.48 b,B	7.79 ± 0.11 b,B	1.97 ± 0.17 a,A	5.95 ± 0.22 b,B	7.49 ± 0.54 c,B	2.01 ± 0.65 ab,A	2.96 ± 0.71 b,A	2.08 ± 0.01 a,A
Fuc^b^	0.41 ± 0.11 ab,B	0.43 ± 0.00 a,A	0.52 ± 0.09 b,B	0.25 ± 0.05 a,AB	0.54 ± 0.19 b,A	0.59 ± 0.02 b,B	0.26 ± 0.04 a,A	0.47 ± 0.12 b,A	0.37 ± 0.02 b,A
Xyl^b^	4.55 ± 1.02 a,C	11.09 ± 0.20 c,C	6.40 ± 0.80 b,C	0.96 ± 0.10 a,B	1.97 ± 0.33 b,B	2.65 ± 0.15 c,B	0.15 ± 0.00 a,A	1.08 ± 0.29 b,A	0.75 ± 0.21 b,A
Man^b^	8.82 ± 0.13 a,A	15.19 ± 0.51 c,A	10.61 ± 0.01 b,A	231.50 ± 15.06 a,C	288.45 ± 9.71 b,C	250.71 ± 10.19 a,C	128.25 ± 0.56 a,B	127.39 ± 13.10 a,B	116.11 ± 22.21 a,B
Gal^b^	138.93 ± 15.46 a,C	541.62 ± 39.06 c,C	402.43 ± 8.18 b,C	46.07 ± 3.88 a,B	55.44 ± 0.15 b,B	88.11 ± 2.49 c,B	26.37 ± 5.49 b,A	24.91 ± 8.74 b,A	13.17 ± 1.04 a,A
GalA^b^	13.49 ± 2.07 a,B	42.45 ± 0.00 b,B	17.35 ± 5.72 a,B	10.92 ± 5.54 ab,AB	10.52 ± 1.11 a,A	17.95 ± 1.60 b,B	7.08 ± 0.56 ab,A	10.51 ± 3.36 b,A	6.78 ± 0.18 a,A
GluA^b^	4.81 ± 1.63 a,C	8.46 ± 3.11 ab,C	10.39 ± 2.93 b,C	1.32 ± 0.04 a,B	1.86 ± 0.25 b,B	2.31 ± 0.66 b,B	1.01 ± 0.18 a,A	0.94 ± 0.03 a,A	0.74 ± 0.26 a,A
Kdo^b^	0.39 ± 0.31 a,AB	0.41 ± 0.06 a,B	0.54 ± 0.03 b,A	0.31 ± 0.13 a,B	0.58 ± 0.08 b,C	0.63 ± 0.23 ab,A	0.13 ± 0.01 a,A	0.22 ± 0.12 a,A	0.56 ± 0.16 b,A
Glc^b^	255.62 ± 51.27 a,C	546.66 ± 67.67 c,B	380.60 ± 69.85 b,C	25.35 ± 4.50 b,B	10.30 ± 3.46 a,A	19.63 ± 4.65 b,B	18.20 ± 0.98 b,A	8.35 ± 1.29 a,A	10.23 ± 2.41 a,A
TMS – (Glc)	189.96 ± 19.88 a,A	674.08 ± 41.93 c,C	515.88 ± 9.02 b,C	300.43 ± 13.11 a,B	374.08 ± 8.04 b,B	379.54 ± 7.59 b,B	167.79 ± 6.97 a,A	172.80 ± 24.70 a,A	143.84 ± 20.20 a,A
TMS – (Glc+Man+ Xyl)	176.59 ± 18.73 a,C	647.79 ± 42.51 c,C	498.86 ± 9.83 b,C	67.97 ± 9.08 a,B	84.55 ± 1.39 b,B	126.17 ± 2.66 c,B	39.38 ± 5.66 b,A	44.32 ± 9.89 b,A	26.98 ± 4.11 a,A
TMS^b^	445.58 ± 71.15 a,C	1220.74 ± 25.74 c,C	896.48 ± 60.82 b,C	325.78 ± 8.61 a,B	385.28 ± 12.11 b,B	399.17 ± 12.24 b,B	185.98 ± 5.99 b,A	181.15 ± 23.41 ab,A	154.07 ± 19.79 a,A
Ara/Gal	0.13 ± 0.00 b,B	0.10 ± 0.01 a,A	0.17 ± 0.03 c,B	0.16 ± 0.01 b,C	0.15 ± 0.02 b,B	0.09 ± 0.03 a,A	0.08 ± 0.01 a,A	0.13 ± 0.07 ab,AB	0.20 ± 0.06 a,B
Rha/GalA	0.31 ± 0.17 a,A	0.21 ± 0.04 a,A	0.56 ± 0.18 a,B	0.24 ± 0.10 a,A	0.67 ± 0.05 c,C	0.49 ± 0.01 b,B	0.33 ± 0.08 a,A	0.34 ± 0.03 a,B	0.36 ± 0.01 a,A
(Ara + Gal)/Rha	45.82 ± 14.46 a,C	73.44 ± 20.08 a,C	55.30 ± 0.89 a,C	24.95 ± 4.05 c,B	9.75 ± 0.54 a,B	11.69 ± 0.86 b,B	13.15 ± 1.51 c,A	8.51 ± 0.48 b,A	6.92 ± 0.23 a,A
RG-II^b^	4.58 ± 0.14 a,A	10.16 ± 0.31 c,A	5.96 ± 0.21 b,A	19.70 ± 3.16 a,B	62.50 ± 13.19 b,B	59.54 ± 11.65 b,C	18.73 ± 5.06 a,B	46.50 ± 9.98 b,B	22.31 ± 0.71 a,B
HL^b^	12.64 ± 2.19 a,B	40.39 ± 0.01 b,B	16.23 ± 5.71 a,C	6.55 ± 4.93 b,AB	0.00 ± 0.00 a,A	5.44 ± 1.50 b,B	2.76 ± 0.34 c,A	0.00 ± 0.00 a,A	2.31 ± 0.05 b,A
PRAG^b^	199.14 ± 22.46 a,C	765.38 ± 59.58 c,C	592.91 ± 3.51 b,C	65.06 ± 5.71 a,B	72.62 ± 2.83 a,B	115.88 ± 2.67 b,B	34.47 ± 7.06 b,A	29.91 ± 12.26 b,A	16.69 ± 0.94 a,A
MP^b^	11.02 ± 0.16 a,A	18.99 ± 0.64 c,A	13.27 ± 0.01 b,A	289.37 ± 18.82 a,C	360.57 ± 12.14 b,C	313.39 ± 12.74 a,C	160.31 ± 0.71 a,B	159.24 ± 16.37 a,B	145.14 ± 27.76 a,B
TSP^b^	227.39 ± 20.57 a,A	834.91 ± 59.26 c,C	628.36 ± 8.98 b,C	380.68 ± 16.44 a,B	495.69 ± 1.78 b,B	494.25 ± 5.26 b,B	216.27 ± 11.08 ab,A	235.66 ± 18.65 b,A	186.44 ± 28.03 a,A

a Average of two measurements. Different letters show statistically significant differences as obtained by Kruskal–Wallis (α = 0.05) with Mann–Whitney pairwise comparison. Lowercase letters compare treatments in the same stage of winemaking. Uppercase letters compare samples of the same treatment in different stages of winemaking.

b 2-*O*MeFuc, 2-*O*-CH_3_-fucose; 2-*O*MeXyl, 2-*O*-CH_3_-xylose; Api, apiose; Ara, arabinose; Rha, rhamnose; Fuc, fucose; Xyl, xylose; Man, mannose; Gal, galactose; GalA, galacturonic acid; GluA, glucuronic acid, Kdo, 2-keto-3-deoxyoctonate ammonium salt, Glc, glucose; TMS, total monosaccharides; RG-II, rhamnogalacturonans type II; HL, homogalacturonans; PRAG, polysaccharides rich in arabinose and galactose; MP, mannoproteins; TSP, total soluble polysaccharide families.

c RM, rosé must; R0, rosé wine at 0 months of aging; R6, rosé wine at 6 months of aging; CP, direct pressing; CM, pre-fermentation maceration; S, pre-fermentative sonication.

Glucose (Glc) was the most prevalent monosaccharide detected in both white (Table 1) and rosé musts (Table 2). Glucose is the prevalent sugar in both the skin and pulp grape berry cell walls.^6,36^ This sugar is the major component of main structural polysaccharides from the grape cell walls such as cellulose and hemicellulosic xyloglucans, arabinoglucans, and mannans. Therefore, the high Glc content could be due to the partial solubilization of these components and to the solubilization of complexes between them and pectic polysaccharides.^6^ In fact, Glc accounted for 91%, 94%, and 94% of the total content of monosaccharides in WM-CP, WM-CM, and WM-S, respectively (Table 1). The content of Glc in rosé must was significantly lower than those obtained in white ones, representing 57%, 45%, and 42% of the total content of monosaccharides in RM-CP, RM-CM, and RM-S, respectively (Table 2), although it was in the range obtained for Tempranillo grapes.^6^ The differences observed in the Glc content in white and rosé musts could be due to the physicochemical and biochemical differences between the cell walls of the different grape varieties. Ortega-Regules et al.^37^ observed that Monastrell grapes showed the highest amount of cell wall material compared with Syrah and Cabernet Sauvignon grapes. The largest amount of Monastrell cell wall structure probably hindered the solubilization of Glc from the cellulosic or hemicellulosic xyloglucans or arabinoglycans or mannans or glucans into the must. According to our knowledge, there are no studies about the cell wall structure of *var*. Viogner. The higher Glc content in the Viogner must suggested a lower amount of cell wall material, therefore, a lower thickness cell wall.

As observed previously in must,^24,25,38^ after Glc, galactose (Gal), arabinose (Ara), and galacturonic acid (GalA) were the three most prevalently glycosyl residues of must polysaccharides (mean value of 4.97%, 0.53%, and 0.54%, in white musts, respectively; and 40.15%, 4.56%, and 2.81% in rosé must, respectively) (Tables 1 and 2). This composition was attributed to the presence of pectic polysaccharides (polysaccharides rich in arabinose and galactose, PRAG, and homogalacturonans, HL) in the must. The highest percentage of Gal, Ara, and GalA in rosé musts was due to lower Glc content compared with white musts.

The presence of xylose residues (Xyl) (mean value of 0.17% and 0.88% in white and rosé must, respectively) suggested that traces of hemicelluloses might be solubilized from the grape berry cell walls.^39^ Rhamnose (Rha), glucuronic acid (GluA), and fucose (Fuc) were also detected in smaller amounts in white (mean value of 0.16%, 0.21%, and 0.03%, respectively) and rosé must (0.75%, 0.98%, and 0.06%, respectively). Rha and GluA are the components of the pectic polysaccharides rich in arabinose and galactose (PRAG), and Rha and Fuc can come from the pectic polysaccharides RG-I or RG-II in the case of Rha,^40^ or only from RG-II in the case of fucose.^41^ The existence of several rare sugars, such as apiose (Api), Kdo (2-keto-3-deoxyoctonate ammonium salt), 2-*O*-methyl-fucose (2-*O*MeFuc), and 2-*O*-methyl-xylose (2-*O*MeXyl) (mean value <0.1% in both white and rosé musts), indicated the presence of the RG-II polysaccharide family.^33^ The identification of mannose residue in must (mean value of 0.25% and 1.47% in white and rosé musts, respectively) was attributed to mannoproteins (MP) of endogenous yeast cell walls^6^ or to mannans or xyloglucans.^1,42,43^

White must from pressing treatment (CP) showed lower content of monosaccharides (except Api and Rha), total monosaccharides (TMS), total monosaccharides less glucose (TMS – Glc), total monosaccharides less the sum of glucose, mannose, and xylose [TMS – (Glc+Man+ Xyl)], RG-II, PRAG, and TSP respect to CM and S musts (Table 1). CP rosé must also show the lowest content of most monosaccharides and polysaccharide families (Table 2). These results confirmed that direct pressing of crushed-destemmed grapes was the least intense treatment for the disruption of the grape berry cell wall polysaccharides, regardless of the grape variety used.

When the cold maceration (CM) pre-fermentation treatment was applied to the crushed-destemmed grapes, the white musts showed higher concentrations of Gal, Glc, TMS, PRAG, and TSP compared to the musts obtained with ultrasonic maceration (S). White must from S treatment had higher Ara, 2-*O*MeFuc, Man, RG-II, and MP or mannan content (Table 1). The biggest differences in the content of monosaccharide and polysaccharide families were observed between the rosé CM and the rosé S musts (Table 2). In fact, CM rosé must show the highest content of 2-*O*MeFuc, 2-*O*MeXyl, Xyl, Man, Gal, GalA, Glc, TMS – (Glc), TMS – (Glc+Man+ Xyl), TMS, RG-II, HL, PRAG, MP, and TSP. These results suggested, on the one hand, that the longer pre-fermentation maceration time applied with the CM technique compared to the short ultrasonic maceration was key in the extraction of polysaccharides from the grape to the must. On the other hand, these results also indicated that the intensity of cell wall degradation caused by the CM and S treatments depended on the grape variety.

Despite the rigidity and firmness of Monastrell cell wall structure,^37^ the action of native pectinases during the cold maceration of Monastrell must in contact with its skin before fermentation, probably, was greater than in the Viognier must/skin maceration. This could explain the greater release of pectins bound to the Monastrel skin. The degree of ripeness of the Monastrell and Viognier grapes was not high, which favored the effect of endogenous enzymes in the degradation of grape cell walls during pre-fermentative cold maceration. Literature indicates the greatest effect of enzymes when they are used during the vinification of grapes with a less advanced degree of maturation, possibly due to the lower degradation that occurs naturally in the structures of the grape with higher maturation.^44^

The disruption of the grape berry cell wall polysaccharides caused by the sonomechanical effect of ultrasounds was more intense in the release of Ara, 2-*O*MeFuc, Man, RG-II, and MP or manans in Viognier than in Monastrell musts. In rosé must, only Api and Kdo significantly increased (Tables 1 and 2). Martínez-Lapuente et al.^45^ pointed out that ultrasound treatment showed a greater effect of deconstructing the polysaccharide network of grape cells, especially when more mature grapes were used. The greater effect of sonication on the extraction of RG-II and mannans or MP in the must was probably due to the lower thickness of the cell wall of Viogner grapes.

PRAG was the most prevalent polysaccharide family in both white and rosé musts, ranging from 89%, 89%, and 84% of TSP in WM-CP, WM-CM, and WM-S, respectively, to 88%, 92%, and 94% in RM-CP, RM-CM, and RM-S, respectively. Several authors^24,25,38^ have observed that arabinogalactans (AGP) were the main polysaccharides released from grapes after crushing and pressing, and it was concluded that these molecules are soluble in plant tissues, requiring less severe techniques for extraction.^36^

The proportion of HL in white musts ranged from 6%, 5%, and 7% in WM-CP, WM-CM, and WM-S, respectivey, to 6%, 5%, and 3% in RM-CP, RM-CM, and RM-S, respectively, in rosé musts. Similar proportions were observed for MP or mannans, which represented only a small percent of total polysaccharide families (4%, 3%, and 5% in the WM-CP, WM-CM, and WM-S, respectively; and from 5%, 2%, and 2% in the RM-CP, RM-CM, and RM-S, respectively).

RG-II presented the lowest amounts ranging from 2% of total soluble polysaccharides in the WM-CP, 3% in the WM-CM, and 5% in the WM-S musts. In rosé musts, RG-II represented 2% in the RM-CP, and 1% in the RM-CP and RM-S of total polysaccharide families. These results agreed with those other authors, who indicated that RG-II is more tightly bound to cell walls than AGP and therefore needs a longer period of maceration to solubilize.^6^

The ratios arabinose to galactose (Ara/Gal), rhamnose to galacturonic acid (Rha/GalA), and arabinose plus galactose to rhamnose (Ara+Gal/Rha) were calculated to better understand the structure of polysaccharides.

The Ara/Gal ratio is characteristic of the PRAG-like structures.^1,2^ Pre-fermentative sonication treatment significantly increased the Ara/Gal ratios compared to CP and CM treatments in white and rosé musts (Tables 1 and 2), indicating that arabinose containing polysaccharides had been extracted from cell walls under sonication treatment. Martínez-Lapuente et al.^25^ also observed a significant increment in the Ara/Gal ratio when high-power ultrasound was applied to crushed Monastrell grapes. The relative richness of polysaccharides rich in homogalacturonans versus rhamnogalacturonans can be deduced from the Rha/GalA ratio.^42^ No significant differences were observed between treatments (Tables 1 and 2). The Ara + Gal/Rha ratio was used to estimate the relative importance of the neutral side-chains to the rhamnogalacturonan backbone, as most of the arabinose and galactose are associated with pectin hairy regions.^18^ Therefore, the highest ratio observed in CM white must might suggest that the rhamnogalacturonan chains in CM must carry more neutral lateral chains.

The transformation of must to wine at the time of bottling (0 months of aging) involved many changes on the glycosyl composition and polysaccharide profile. Regardless of treatment and grape variety, TMS content decreased in wines mainly due to a decrease in Glc content (Table 1 and 2), suggesting a precipitation of glucose by the action of endogenous enzymes and/or the content of ethanol formed during alcoholic fermentation.^25^ Glc, which is not a component of pectic polysaccharides, was attributed to polysaccharides of yeasts and/or bacteria in wines.^7,17^ The fermentation process (without the presence of the skin tissues) involved a depectinization or degradation of the pulp cell walls, composed, according to Gao et al.,^46^ of abundant RG-I pectin side chains (linear and branched arabinans) and extensin glycoprotein. The effect of the depectinization of pulp tissues during alcoholic fermentation on the content of pectic polysaccharide families in wines was similar in both grape varieties. Thus, PRAG and HL content significantly decreased in white and rosé wines at the time of bottling (0 months of aging) compared to initial must, while RG-II significantly increased.

The decrease in PRAG content during alcoholic fermentation of white and rosé musts was not observed in red wines, where the alcoholic fermentation occurred with skin contact; on the contrary, literature describes a significant increase in the PRAG content in red wine compared to the initial must.^24,25^ The absence of skin tissues during the alcoholic fermentation of white and rosé musts had, therefore, consequences on the polysaccharide composition of the resulting wines. First, there was not a release of PRAG from the cell walls of the skin tissues to the wine, which explains the lower PRAG content in white and rosé wines compared to red wines.^36,47^ Second, the absence of skin tissues during alcoholic fermentation did not compensate for the loss of PRAG, which is probably caused by the lower solubility of PRAG due to the presence of ethanol formed during the alcoholic fermentation. As previously discussed, PRAG was the most soluble polysaccharide family in an aqueous medium, such as white and rosé musts, while it was not in wines (data discussed later). The progressive depectinization of the skin tissues during maceration-fermentation^48^ causes the tissues to act as a releasing source of pectic polysaccharides to the must, increasing its soluble content in young red wine.^24,45^ Vidal et al.^36^ determined that 75% of the grape berry walls originates from the skin tissue. In the same way, a significant increase in RG-II from must to wine (0 months of aging) elaborated without the presence of skin tissues was also observed in red wines.^24^ However, RG-II content in white wines from Viogner and rosé from Monastrell at the time of bottling was lower than that of the young red wines from Monastrell.^24,25^ Guadalupe and Ayestarán^6^ observed that RG-II needed more time to solubilize, as it was more tightly bound to the cell wall matrix of grape cell walls, compared to the rapid solubilization of the PRAG that began from the beginning of the maceration. MP displayed similar behavior to RG-II. The liberation of yeast mannoproteins during alcoholic fermentation increased the MP content in white and rosé wines (Tables 1 and 2).

Passing from must to wine at the time of bottling (0 months of aging) produced changes in the polysaccharide characteristic ratios. Ara/Gal ratio increased in white and rosé wines, except for R0-S. Rha/GalA evolution was different in white and rosé wines. The ratio decreased in W0-CP, and in W0-S and W0-CM wines remained constant, while it increased in R0-CM and remained constant in R0-CP and R0-S. In general, a significant decrease in Ara + Gal/Rha ratio for white and rosé wines was observed (Tables 1 and 2).

White wines at the time of bottling (0 months of aging) from direct pressing treatment (CP) showed lower values of TMS, TMS – (Glc), RG-II, HL, PRAG, and TSP than white wines CM and S, except for RG-II content, which was like W0-CM wine (Table 1). Similar results were observed in R0-CP wine (Table 2). PRAG content was similar between R0-CP and R0-CM wines, while R0-CP and R0-S wines showed the highest values of HL (Tables 1 and 2). In general, these results indicated that CP treatment was the least intense for the disruption of the grape berry cell wall polysaccharides and produced white and rosé wines with lower TMS and TSP content at the time of bottling.

CM white and rosé musts showed higher TMS, TSP, and PRAG content than S musts. However, these differences were not observed in the wines at the time of bottling (Tables 1 and 2). TMS, TMS – (Glc), and TSP content was similar between W0-CM and W0-S wines, and between R0-CM and R0-S wines. W0-S wine showed higher values of Rha, Gal, GalA, TMS – (Glc+Man+ Xyl), HL, and PRAG than W0-CM wine. In the same way, R0-S wine showed higher values of Rha, Gal, GalA, TMS – (Glc+Man+ Xyl), HL, and PRAG than R0-CM wine. These results indicated that short ultrasonic maceration of the grapes caused changes in the cell wall structure of the pulp tissues during alcoholic fermentation, and increased the solubility of polysaccharides to a greater extent than the long maceration of CM. This fact produced a greater release of PRAG and HL, regardless of the grape variety and its low degree of maturation. Therefore, ultrasonic pre-fermentative treatment of the grapes positively affected wine colloidal properties, potentially increasing polysaccharide solubility. Results suggested that ultrasound maceration treatment of grapes could be used as a new tool to increase the content of pectic polysaccharides in wines at time of bottling.

However, the RG-II content was only significantly higher in W0-S wines. This result suggested that the lower thickness of the cell wall of Viogner favored that US treatment progressively solubilized more RG-II during alcoholic fermentation. In summary, the sonication treatment of the grapes, with shorter pre-fermentative maceration time than CM,^31^ improved the progressive depectinization of the cell walls of pulp tissues during alcoholic fermentation. This depectinization was more intense in the Viogner grape variety, which would indicate a lower thickness of its cell wall compared to the Monastrell grape.

MP content of W0-CM wines was similar to that of the W0-S, and higher than that of W0-CP wines. R0-CM wines showed the highest MP values. These results indicated that the application of the techniques influenced the degradation of the yeast cell walls, probably because the treatments caused different degrees of turbidity in the musts during alcoholic fermentation. Previous studies have shown that the release of MP from yeast into the wine matrix depends on both the yeast strain^49^ and the turbidity of the must.^50^

As a result, wines at the time of bottling were largely dominated by MP (ranging from 72%, 74%, and 67% in W0-CP, W0-CM, and W0-S, respectively, and from 76%, 73%, and 63% in R0-CP, R0-CM, and R0-S, respectively), followed by PRAG (21% in all white wines at 0 months of aging and 17%, 15%, and 23% in R0-CP, R0-CM, and R0-S, respectively), RG-II (ranging from 6%, 5%, and 8% in W0-CP, W0-CM, and W0-S, respectively, and from 5%, 13%, and 12% in R0-CP, R0-CM, and R0-S, respectively), and HL (ranging from 1%, 1%, and 3% in W0-CP, W0-CM, and W0-S, respectively, and from 2%, 0%, and 1% in R0-CP, R0-CM, and R0-S, respectively). These proportions were not in agreement with those described for red wines,^2,6^ in which pectic polysaccharides are liberated from grape skins and pulp during maceration-fermentation.

In general, the content of glycosyl residues, TMS, TMS – (Glc), TMS – (Glc+Man+ Xyl), RG-II, HL, PRAG, MP, and TSP remained constant or decreased in the wines after 6 months of aging. These results agree with those obtained by other authors during the aging of Carignan noir wines.^33^

In white wines, TSP decreased 45%, 21%, and 41% in W6-CP, W6-CM, and W6-S wines, respectively (Table 1). In rosé wines, the decrease was more pronounced, with 43%, 52%, and 62% in W6-CP, W6-CM, and W6-S wines, respectively (Table 2). The greater decrease of TSP in rosé wines may be a consequence of the greater formation of complexes between the polysaccharides and other wine compounds.^7^ Jones-Moore et al.^4^ observed that polysaccharides participate in the formation of colloidal particles through interactions with wine tannins and proteins, which affect the clarity and stability of finished wines and thus the organoleptic properties of aged wines.^47^ No clear effect of the pre-fermentative treatment on the evolution of polysaccharides in wines was found. Future studies should be carried out on the organoleptic consequences caused by the polysaccharide evolution during bottle aging of Monastrell rosé and Viogner white wines.

A decrease of the Ara/Gal ratio was observed in white wines from CP, CM, and S treatments. This decrease was only observed in CP rosé wines, while it was maintained in CM rosé wines and decreased in S rosé wines. These results suggested that during bottle aging, in general, the terminal arabinose residues in wines were removed. This reduction of arabinose residues indicated a dearabinosylation of arabinogalactan-proteins. The different evolution in the Ara/Gal ratio among wines may influence the PRAG physicochemical properties and thus modify the final colloidal equilibrium. No clear changes in Rha/GalA ratios were observed during bottle aging. In general, the Ara + Gal/Rha ratio remained constant in white wines during bottle aging, while it decreased in rosé wines.

After six months of bottle aging, white wines from CM treatment had the highest PRAG, TSP, and MP content, while R6-CM wines showed the highest RG-II content. CP rosé wines at 6 months of bottle aging showed the highest HL content while white CP wines showed high RG-II contents. RG-II content was similar in W6-CP and W6-S wines. These very different results in the evolution of pectic polysaccharides suggested that the influence of pre-fermentative treatments applied to grapes did not dominate of polysaccharides during aging.

MP were the majority polysaccharides in both white and rosé wines after 6 months of bottle aging, ranging from 68%, 77%, and 74% of TSP in W6-CP, W6-CM, and W6-S, respectively, and from 74%, 68%, and 78% in R6-CP, R6-CM, and R6-S, respectively. PRAG were the second largest polysaccharides in white wines, and ranged from 21%, 21%, and 16% of TSP in W6-CP, W6-CM, and W6-S, respectively, followed by RG-II (9%, 2%, and 8% of TSP in W6-CP, W6-CM, and W6-S) and HL (3%, 0%, and 3% of TSP in W6-CP, W6-CM, and W6-S, respectively). However, in rosé wines, similar proportions of RG-II and PRAG were found. In fact, RG-II showed 9%, 20%, and 12% of TSP in R6-CPD, R6-CM, and R6-S, respectively, and PRAG proportions were 16%, 13%, and 9% in R6-CPD, R6-CM, and R6-S, respectively.

### 3.2 Differentiation of White and Rosé Must and Wines According to Monosaccharide Composition and Polysaccharide Families

**Figure 1:**
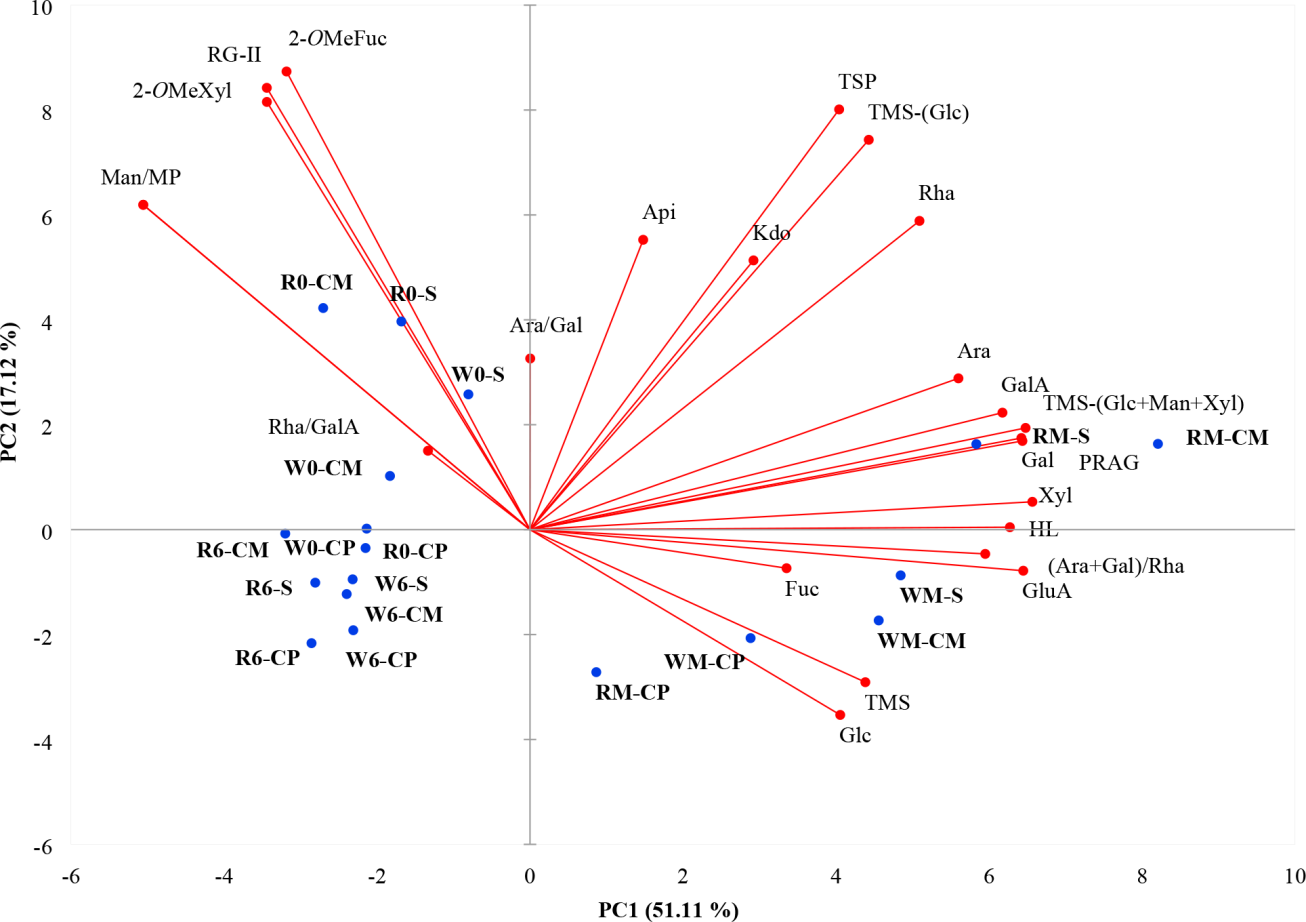
Principal component analysis (PCA) of the wines performed with monosaccharides and polysaccharide families concentration. 2-*O*MeFuc, 2-*O*-CH_3_-fucose; 2-*O*MeXyl, 2-*O*-CH_3_-xylose; Api, apiose; Ara, arabinose; Rha, rhamnose; Fuc, fucose; Xyl, xylose; Man, mannose; Gal, galactose; GalA, galacturonic acid; GluA, glucuronic acid, Kdo, 2-keto-3-deoxyoctonate ammonium salt, Glc, glucose; TMS, Total monosaccharides; RG-II, rhamnogalacturonans type II; HL, homogalacturonans; PRAG, polysaccharides rich in arabinose and galactose; MP, mannoproteins; TSP, total soluble polysaccharide families. WM, white must; W0, white wine at 0 months of aging; W6, white wine at 6 months of aging; RM, rosé must; R0, rosé wine at 0 months of aging; R6, rosé wine at 6 months of aging; CP, direct pressing; CM, pre-fermentation maceration; S, pre-fermentative sonication.

To classify the different treatments, grape variety, and vinification stages, PCA was performed using all the data of the musts and wines. The results are shown in Figure 1. Principal component (PC1) explained 51.11% of the variance, and PC2 explained 17.12% of the variance, representing a 68.23% of the total variance.

PC1 was strongly correlated with Ara, Rha, Xyl, Man, Gal, GalA, GluA, (Ara + Gal)/Rha, HL, PRAG, MP, and TMS – (Man + Glc + Xyl). PC2 was strongly correlated with 2-*O*MeXyl and RG-II. PC allowed differentiating between winemaking stages, grape variety, and pre-fermentation treatments.

The variables mainly associated with PC1 allowed differentiation of the samples by the winemaking stage. Musts were sited on the positive side of PC1, while wines at 0 and 6 months of aging were located on the negative side of PC1. Therefore, both the alcoholic fermentation and bottle aging affected the monosaccharide and polysaccharide profile. This fact was mainly due to the increase of Man and MP released by yeast during alcoholic fermentation (as previously discussed in section 3.1), from must to wines, and to the decrease of Ara, Rha, Xyl, Gal, GalA, GluA, (Ara + Gal)/Rha, HL, PRAG, and TMS – (Man + Glc + Xyl), compounds associated with PC1. Rosé musts from CM and S treatments were widely separated from the other musts and they were in the most positive part of PC1. They were highly correlated to Ara, Gal, PRAG, TMS – (Man + Glc + Xyl) due to its higher content compared to other musts (Tables 1 and 2).

WM-S and WM-CM, which were in the negative part of PC2, were positively correlated with GluA, (Ara + Gal)/Rha, TMS, and Glc. WM-CP and RM-CP were widely separated from the other musts. WM-CP and RM-CP were located in the most negative part of PC2 due to its lower monosaccharide and polysaccharide content compared to musts elaborated with CM and S treatments (Tables 1 and 2). Except for CP samples, wines at 0 months of aging were located in the fourth quadrant, which was mainly defined by RG-II, MP, 2-*O*MeFuc, 2-*O*MeXyl, and Rha/GalA. A differentiation between rosé and white wines was observed at 0 months of aging. Rosé wines from CM and S treatments appeared much farther to the origin than white wines because they showed higher contents of RG-II and MP than the white wines CM and S (Tables 1 and 2). However, no differentiation by pre-fermentative treatment and grape variety was observed in wines after 6 months of aging. Therefore, all wines at 6 months of aging were located in the third quadrant and were inversely correlated with monosaccharides and total polysaccharides content.
